# Mitophagy as a therapeutic target for exercise-induced fatigue: modulation by natural compounds and mechanistic insights

**DOI:** 10.3389/fphys.2025.1664909

**Published:** 2025-10-31

**Authors:** Miao Yu, Xiujuan Jiang, Yingxin Zhang, Wensi Zhang, Tianlong Wang, Jialin Wang, Junwei Shao, Lixin Zhang, Yiting Sun, Xianglong Meng, Xiaohong Li, Xianjun Liu

**Affiliations:** ^1^ College of Biological and Food Engineering, Jilin Engineering Normal University, Changchun, China; ^2^ University of Waterloo, Waterloo, ON, Canada; ^3^ Department of Gastrointestinal Colorectal and Anal Surgery, The China-Japan Union Hospital of Jilin University, Changchun, China; ^4^ Department of Gastroenterolgy & Hepatology, China-Japan Union Hospital, Jilin University, Changchun, China

**Keywords:** mitophagy, exercise-induced fatigue, natural compounds, therapeutic targets, mitophagy pathways

## Abstract

Exercise-induced fatigue is closely associated with mitochondrial dysfunction, and mitophagy plays a critical role in maintaining mitochondrial homeostasis by clearing damaged mitochondria and reducing oxidative stress. This review systematically summarizes current evidence on the regulatory mechanisms of mitophagy in exercise-induced fatigue, particularly through pathways such as PINK1/Parkin, BNIP3/Nix, FUNDC1, and AMPK, and examines how natural compounds including sulforaphane, *Rhodiola crenulata*, ginseng, modulate these pathways to alleviate fatigue. These findings suggest the presence of mitophagy threshold in different models and highlight its potential as a therapeutic target for fatigue management. Ultimately, this review proposes novel strategies for developing natural anti-fatigue agents based on mitophagy regulation, while underscoring the need for further mechanistic studies in diverse physiological and pathological settings.

## 1 Pathophysiological characteristics of exercise-induced fatigue

Exercise-induced fatigue, defined as the inability to maintain a specific level or intensity of physical activity (Rosenthal et al., 2008; O'Sullivan et al., 2018), represents a physiological warning signal following excessive exertion rather than a pathological condition (Li et al., 2022a). Its research scope has expanded from athletic performance to broader health management.

Studies classify fatigue mechanisms into three categories, depletion of activity-required substrates, accumulation of metabolic byproducts such as lactic acid, and oxidative stress caused by free radicals (Jin and Zheng, 2008). Substrate depletion triggers the conversion of fats and proteins into energy substrates, which must be transformed into ATP and creatine phosphate for effective utilization. Excessive lactic acid accumulation impairs muscular contraction and relaxation by inhibiting fructose-1,6-bisphosphate aldolase, thereby impeding ATP synthesis (Li and Zhao, 2017; Melvin, 1998). During exercise, overproduction of free radical damages proteins and DNA, impairs organelles, decreases cell membrane fluidity, disrupts the tricarboxylic acid cycle, and ultimately induces fatigue (Yakes and Van Houten, 1997; Davies et al., 1982; Jackson and Farrell, 1993). Furthermore, reactive oxygen species (ROS) accumulation after high-intensity exercise can cause myocardial lipid peroxidation, threatening long-term health (Mu, 2023; You et al., 2011).

For athletes, fatigue is a core factor limiting competitive performance, as excessive fatigue may lead to muscle damage, metabolic dysregulation, and impaired organ dysfunction (Yang, 2016). Understanding fatigue mechanisms can help optimize athletic training programs, such as targeting mitophagy to remove damaged mitochondria, and provide strategies for scientific anti-fatigue research. Effectively management of exercise-induced fatigue requires enhancing the body’s antioxidant capacity.

Current anti-fatigue products aim to rapidly restore physical strength through direct ATP precursor supplementation, reduce oxidative stress by neutralizing free radicals, and delay subjective fatigue via central nervous system stimulation. However, these approaches fail to address root causes such as low mitochondrial oxidative phosphorylation efficiency. Long-term use may disrupt endogenous antioxidant system balance and mask true physiological strain, increasing the risk of exercise-related injuries.

## 2 Mitophagy: a central mechanism in cellular homeostasis and disease

Mitophagy, a selective form of autophagy responsible for removing damaged mitochondria, is essential for maintaining cellular energy homeostasis and viability (Onishi et al., 2021). This process is a key component of the mitochondrial quality control system, which also includes biogenesis, fusion, and fission (Yoo and Jung, 2018). Autophagy participates in multiple physiological processes, including organismal development, adaptive immune system function, and cellular energy homeostasis maintenance.

Research indicates that mitophagy is closely linked to numerous diseases, playing a crucial role in neurodegenerative disorders (Li et al., 2023), cardiovascular conditions (Ajoolabady et al., 2022), bone diseases (Zeng et al., 2022), and cancer (Panigrahi et al., 2020).

Neurodegenerative diseases-characterized by misfolded protein accumulation and mitochondrial dysfunction (Ma et al., 2021)-include prion diseases (Gao et al., 2020), Alzheimer’s disease (Li et al., 2022b), Parkinson’s disease (Jiang et al., 2022), and Huntington’s disease (Zilocchi et al., 2018; Khalil et al., 2015; Franco-Iborra et al., 2021), all associated with impaired mitophagy.

Cardiovascular conditions such as hypertension (Ding et al., 2022), atherosclerosis (Xi et al., 2022), ischemic heart disease (Siddall et al., 2013), and heart failure (Feng et al., 2018)is caused by mitochondrial dysfunction. Notably, exercise can mitigate heart failure-a severe condition with high mortality. Further investigation into exercise-induced mitophagy mechanisms and optimal intensity regulation for safe, effective induction may yield valuable insights for cardiovascular disease treatment and intervention (Zhang et al., 2022).

Abnormal mitophagy may also contribute to bone diseases including osteoporosis, osteoarthritis, and osteosarcoma. As a therapeutic target for such conditions, mitochondrial dynamics informs bone disease treatment research (Gao et al., 2021; Yao et al., 2019; Gorska-Ponikowska et al., 2021).

Mitophagy further correlates with cancer development. In gastric carcinogenesis, progressive autophagy downregulation coupled with increasing glycolysis during the transition from benign gastric disease to malignancy ultimately facilitates cancer occurrence (Giatromanolaki et al., 2013).

To maintain cellular function and homeostasis, dysfunctional mitochondria require timely clearance. Unrepaired damaged mitochondria cause energy deficits that impair physiological activities. Through sophisticated autophagic mechanisms, cells identify and eliminate these organelles, preserving energy production efficiency and cellular vitality. Exercise-induced mitophagy represents a current research focus, with ongoing discoveries of mitophagy receptors and proteins regulating these processes.

## 3 Key mitophagy pathways implicated in exercise fatigue regulation

### 3.1 PINK1/Parkin: dual roles in exercise contexts

The PINK1/Parkin pathway plays a crucial role in mitochondrial quality control. PINK1, a serine/threonine kinase, accumulates on damaged mitochondrial membranes and recruits the E3 ubiquitin ligase Parkin to initiate mitophagy (Tian et al., 2015; Tatsuta and Langer, 2008; Park et al., 2006; Clark et al., 2006). In exercise contexts, high-intensity activity inhibits proteasomal degradation of PINK1, leading to its accumulation and subsequent pathway activation, which peaks around 12 h post-exercise—coinciding with maximal mitochondrial damage (Botella et al., 2018) (Shang et al., 2018).

#### 3.1.1 Natural compounds inhibiting PINK1/Parkin in exercise-induced fatigue

Multiple studies demonstrate that natural compounds and drugs modulate the PINK1/Parkin-mediated mitophagy pathway. Sulforaphane (SFN)-exhibiting antioxidant (Ma et al., 2023), anticancer (Kamal et al., 2020), anti-aging (Santín-Márquez et al., 2019), and antiviral (Ordonez et al., 2022) properties. *Rhodiola crenulata*, a Tibetan Crassulaceae plant, contains the primary active compound kaempferol with anti-inflammatory (Pu et al., 2020), neuroprotective (Zhang et al., 2019), radioprotective (Arora et al., 2005), and anticancer effects (Ravi et al., 2025). Guo et al., (2022) and Hou et al. (2020) investigated SFN and *Rhodiola crenulata* oral liquid effects on PINK1/Parkin signaling, exercise-induced mitophagy, and skeletal muscle fatigue. Despite different exercise models-treadmill vs. and weighted swimming, both studies reported reduced skeletal muscle damage, enhanced antioxidant capacity. Hou et al. additionally measured total antioxidant capacity and Na^+^-K^+^-ATPase activity, and attenuated fatigue through PINK1/Parkin-mediated mitophagy inhibition. Wang et al. (2023) subsequently found that a ginseng compound formula similarly inhibits PINK1/Parkin-mediated mitophagy to influence fatigue. Unlike prior studies, Wang et al. observed dose-dependent effects on loaded swimming time, 4.16 and 16.66 mL/kg doses significantly prolonged swimming versus controls, while 8.33 mL/kg showed no statistical difference-warranting further investigation into this anomalous result. Yuan et al. (2022) employed a fatigue-with-myocardial-injury model investigated Astragalus Shengmai Decoction-derived from Shengmai Powder and containing *Astragalus*, *Codonopsis*, *Ophiopogon*, *Schisandra*, and *Southern Schisandra*-which tonifies Qi, restores pulse rhythm, nourishes Yin, and promotes fluid production, enhancing myocardial hypoxia tolerance while reducing oxygen consumption (Qu and Hao, 2017; Jiang et al., 2021), confirmed Astragalus Shengmai Decoction’s inhibitory effect on PINK1/Parkin expression.

#### 3.1.2 Divergent roles of the PINK1/Parkin pathway in chemotherapy-induced fatigue

**FIGURE 1:**
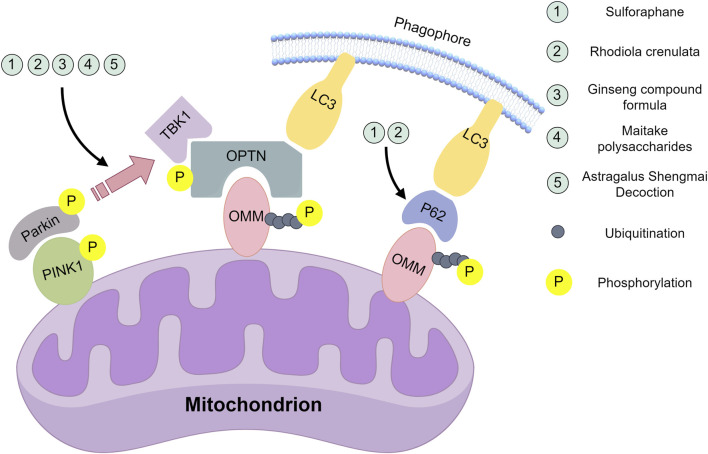
Natural compounds act on the key nodes of the PINK1/Parkin signalling pathway. Sulforaphane: Inhibition of mRNA and protein expression of PINK1 and Parkin; inhibition of PINK1/Parkin-dependent mitochondrial ubiquitination; downregulation of p62 protein levels. Rhodiola crenulate oral liquid: Inhibition of mRNA and protein expression of PINK1 and Parkin; inhibition of PINK1/Parkin-dependent mitochondrial ubiquitination; downregulation of LC3-II/LC3-I ratio and p62 protein levels. Ginseng compound formula: Inhibit the mRNA expression of PINK1 and Parkin. Astragalus Shengmai Decoction: Inhibit the protein expression of PINK1 and Parkin. Maitake polysaccharides:Promote the protein expression of PINK1 and Parkin. This figure was created by Figdraw (www.figdraw.com).

Lei et al. (2019) employed a chemotherapy-induced fatigue (CIF) model,. Lei et al. utilized Maitake polysaccharides extracted from fruiting bodies, possessing immunomodulatory, antitumor, anti-HIV, antihypertensive, anti-fatigue, antioxidant, and pro-apoptotic properties in hepatocellular carcinoma (Xiao et al., 2022; Zhao et al., 2023). Demonstrated impaired mitophagy via PINK1/Parkin downregulation in CIF, which Maitake polysaccharides ameliorated by upregulating these proteins. This discrepancy may stem from model differences (Figure 1).

In summary, natural compounds such as SFN, ginseng, astragalus, and RC alleviate exercise-induced fatigue by inhibiting PINK1/Parkin-mediated mitophagy. However, the mechanism of Maitake polysaccharides is fundamentally different: it upregulates suppressed PINK1/Parkin expression to restore autophagic homeostasis in chronic fatigue models. This seemingly contradictory phenomenon highlights the specificity of mitophagy under different stressors. To thoroughly investigate this phenomenon, we must extend beyond PINK1/Parkin itself and consider upstream/downstream targets for deeper exploration of natural product mechanisms. SFN and RC may neutralize excess ROS generated during early exercise through their potent antioxidant properties, thereby reducing mitochondrial damage signals upstream and preventing excessive activation of the PINK1/Parkin pathway. The cardiomyopathy-enhancing effects of Astragalus Shengmai Decoction, such as improving myocardial hypoxia tolerance and reducing oxygen consumption may collectively lower relative hypoxia levels during exercise, indirectly mitigating mitochondrial damage. Maitake polysaccharides might regulate upstream signals of the PINK1/Parkin pathway, functionally restoring mitochondrial self-renewal capacity. In the future, research should be devoted to revealing whether these natural products are multi-target synergistic in mitophagy or whether there is an initial and core target, further analyze the mitophagy threshold in different models, and explore the precise intervention strategy of PINK1/Parkin pathway.

### 3.2 Nix/BNIP3: bidirectional regulatory factor in exercise stress

Nix (BNIP3L), a pro-apoptotic mitochondrial outer membrane protein (Liu et al., 2019), shares 56% cDNA homology with BNIP3 (Ashrafi and Schwarz, 2013). Both are Bcl-2 family members involved in mitophagy. Nix-mediated mitophagy occurs during erythrocyte maturation (Sandoval et al., 2008), while hypoxia upregulates Nix and BNIP3 to induce mitophagy (Zhang et al., 2008). BNIP3 also regulates alternative mitophagy pathways by preventing PINK1 degradation, leading to PINK1 accumulation and subsequent PINK1/Parkin-mediated mitophagy (Zhang et al., 2016).

#### 3.2.1 Positive activation of mitophagy

Jamart et al. (2013) and Bo et al. (2014) respectively demonstrated that fasted endurance training and hypoxic exercise significantly increase Bnip3 and Nix mRNA expression, indicating enhanced mitophagy. Similarly, Liao et al. (2020) found high-intensity interval training (HIIT) activates myocardial BNIP3 signaling in middle-aged mice, elevating Bnip3/Nix expression, increasing mitochondrial quantity, and improving respiratory function.

#### 3.2.2 Inhibition of excessive mitophagy

**FIGURE 2:**
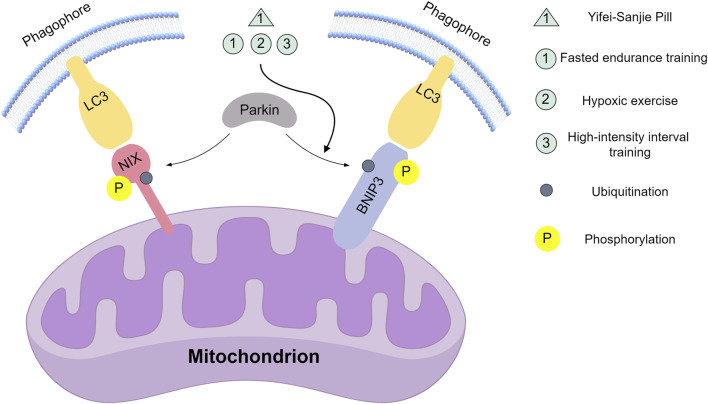
Natural compounds and movement modes act on key nodes of the Nix/BNIP3 signalling pathway. Yifei-Sanjie Pill: Inhibit the expression of BNIP3. Fasted endurance training, Hypoxic exercise, High-intensity interval training: Promote BNIP3 expression. This figure was created by Figdraw (www.figdraw.com).

Ma et al. (2011) observed reduced Bnip3/Nix expression, improved mitochondrial function, and decreased mitophagy following endurance training in mice with alcohol-induced liver injury, suggesting enhanced hepatic oxygen supply. Wu et al. (2022a) studied Yifei-Sanjie pill-a Qi-tonifying, phlegm-resolving formula containing *Uncaria rhynchophylla*, *Bombyx mori pupae*, *Arisaema heterophyllum*, *Lilium brownii*, *Fritillaria thunbergii*, *Pinellia ternata*, *Ganoderma lucidum*, and *Panax quinquefolius* Wu et al. (2023) showing it inhibits BNIP3 pathway-mediated skeletal muscle mitophagy in exhausted tumor-bearing mice (Figure 2).

Collectively, these findings indicate that combining endurance training with Traditional Chinese Medicine (TCM) may effectively regulate mitophagy and enhance functional outcomes. Regarding adaptive activation, fasting-induced endurance training, hypoxic exercise, or HIIT as physiological hypoxia stimuli can upregulate Bnip3/Nix expression. In terms of inhibiting hyperactivation, within the pathological context of alcoholic liver injury, endurance training improves hepatic oxygen supply and systematically reduces oxidative stress, thereby decreasing excessive demand on the Bnip3/Nix pathway. Yifei-Sanjie Pill inhibits BNIP3-mediated hyperautophagy, where multiple herbal components may act as multi-target regulators to stabilize metabolic homeostasis, indirectly modulating BNIP3 expression. Future research should focus on analyzing potential synergistic effects among Yifei-Sanjie Pill’s components and identifying which key ingredients play dominant roles.

### 3.3 FUNDC1: a hypoxia-sensing mitophagy receptor

FUNDC1, a mitochondrial outer membrane receptor, senses hypoxia and initiates mitophagy through dephosphorylation and subsequent binding to LC3 (Mao et al., 2020; Shi, 2018; Wu et al., 2016). This mechanismis essential for the selective removal of damaged mitochondria under low-oxygen conditions.

Electrical pulse stimulation, a non-invasive neuromuscular technique, modulates muscle tone, strength, endurance, circulation, and recovery (Neumann et al., 1982). Gao. (2019) demonstrated its induction of FUNDC1-mediated mitophagy, post-stimulation increases in PGC-1α, COX-I, LC3, and FUNDC1 coincided with p62 reduction. This process activates the AMPK-ULK1 pathway to initiate mitophagy. Separately, Yan et al. (2022) identified Fenugreek Seed extract, which contains galactomannan, steroidal saponins, flavonoids, alkaloids, terpenes, and coumarins (Toshiyuki et al., 2000; Masayuki et al., 1997),as an anti-fatigue agent acting through FUNDC1/LC3B pathway inhibition, independent of PINK1/PARKIN signaling, thereby enhancing rat exercise performance.

These studies clarify FUNDC1’s role and mechanisms in mitophagy, revealing new insights into autophagy regulation. Physical stimuli including electrical pulses (Neumann et al., 1982) activate this pathway to clear damaged mitochondria, while chemical interventions like Fenugreek Seed balance autophagy intensity by modulating pathway activity to alleviate fatigue. Future, research should be committed to identifying specific intervention targets for FUNDC1 regulation, verifying whether fenugreek seed directly acts on FUNDC1 itself or its upstream regulatory factors, and precisely regulating FUNDC1 through the intersection of physical intervention and natural pharmacological chemistry.

### 3.4 AMPK: the cellular energy sensor governing mitophagy

AMPK, an AMP-dependent protein kinase and primary cellular energy sensor, is regulated by AMP levels altered during ATP hydrolysis (Steinberg and Hardie, 2022). It monitors cellular energy and nutrient status (Hardie, 2014) and is activated by natural compounds including curcumin (Wong et al., 2009; Zhan et al., 2015). Exercise excess, hypoxia, oxidative stress, and ischemia activate the AMPK-mediated autophagy pathway, phosphorylating key metabolic and transcriptional regulators while affecting all cellular metabolism branches (Khan et al., 2021). Exercise elevates muscular energy metabolism, modifying AMP levels and consequently AMPK activity (Hancock et al., 2006). AMPK enhances autophagy through TSC2 and Raptor phosphorylation (Inoki et al., 2006). Phosphorylation sites act as molecular switches that precisely regulate the initiation, amplification, and termination of mitophagy by altering protein conformation, activity, or intermolecular interactions. This process involves the coordinated action of multiple signaling pathways, ultimately ensuring the selective clearance of damaged mitochondria and the maintenance of energy homeostasis under stress conditions. Targeting these phosphorylation sites may constitute a promising strategy for managing exercise-induced fatigue in future research.

Current research investigates AMPK-mediated mitophagy using aerobic exercise combined with natural compounds. Yan. (2023) and Dun et al. (2017) demonstrated that curcumin and RC increase AMPK expression, activate mitophagy, and enhance skeletal muscle mitochondrial quality control. Dun et al. further identified RC’s synergistic cardioprotective effect on congenital myocardial injury and myocardial mitochondrial quality. Wang. (2021) compared HIIT and moderate-intensity continuous training (MICT) in high-fat-diet mice, finding both elevated AMPK expression. MICT more effectively enhanced mitophagy, restoring mitochondrial function and maintaining skeletal muscle mitochondrial content. Wang et al. (2021) observed that chronic stress inhibits AMPK signaling, blocking mitophagy and causing gastrocnemius mitochondrial dysfunction. Collectively, aerobic exercise and natural compounds regulate AMPK-mediated mitophagy to improve mitochondrial quality control.

These studies advance understanding of the AMPK-mediated mitophagy pathway, demonstrating the potential of aerobic exercise and natural compounds to enhance mitochondrial quality control. They specifically reveal the superior efficacy of MICT for skeletal muscle mitochondrial function. This advantage may arise because MICT producessustained, mild energy stress that enables AMPK to activate autophagy flux in a more sustainable and non-destructive manner; whereas HIIT may trigger excessive stress that activates more antagonistic or complex signalling, thereby diminishing the net benefit of AMPK-mediated mitochondrial quality control. Curcumin and RC may activate AMPK, thereby driving a series of mitophagy-promoting processes. Future research should focus on identifying the critical thresholds where AMPK and its key downstream targets facilitate adaptive responses and trigger metabolic depletion under different exercise modes. Additionally, it is crucial to determine whether curcumin and RC directly act on AMPK itself or function as upstream kinases.

### 3.5 Additional mediators of mitophagy in exercise fatigue

Gong. (2021) compared mitophagy responses across exercise regimens-moderate-intensity continuous, resistance, and HIIT versus exhaustive exercise alone. All protocols significantly increased LC3II expression versus controls, with the exhaustive-only group showing the highest LC3II levels. This group also exhibited elevated FKBP8 protein expression relative to other exercise modalities.

Fix et al. (2018) demonstrated that skeletal muscle gp130 receptor absence does not impair exercise-induced Beclin-1 expression but mediates mitophagosome formation during oxidative stress.

Huang et al. (2016) further established an inverse correlation between endurance and muscle malondialdehyde levels, confirming astragalus polysaccharides enhance exercise capacity in oxidative stress models by boosting antioxidant enzyme activity and ameliorating mitochondrial dysfunction.

Weichmann et al. (2021) reported Robinia pseudoacacia extract alleviates physical fatigue; its primary component quercetin elevates mitophagy, promotes mitochondrial biogenesis, enhances antioxidant capacity, and improves exercise performance.

However, the reported associations between exercise and induced mitophagy warrant further investigation. Mitophagy stability is essential for metabolic homeostasis, as its dysregulation contributes to various pathologies. Certain factors and natural components enhance autophagy-related protein expression, promoting mitophagy to restore aerobic adaptation and mitochondrial regeneration. In the future, the research should be committed to deeply analyzing the direct molecular targets of natural products such as astragalus polysaccharides and Robinia pseudoacacia extract in regulating mitophagy, exploring the interaction between multiple pathways, and whether other pathways will be activated compensatorily after a certain pathway is decreased under specific conditions.

Current anti-fatigue products face significant efficacy limitations. Energy supplements and antioxidants provide symptomatic relief without fundamental correction, as their mechanisms lack deep regulation of core fatigue factors like mitochondrial dysfunction and oxidative balance. Mitophagy-targeting products offer distinct advantages, by enabling cells to eliminate damaged mitochondria, they maintain mitochondrial quality control at its source, reduce oxidative stress accumulation, and restore energy homeostasis. Compared to conventional products, these novel interventions demonstrate enhanced specificity, achieving true “repair and regeneration” effects (Table 1).

**TABLE 1:** Analysis of exercise fatigue and mitochondrial autophagy.

Document number	Medicines/natural ingredients	Research model	Main conclusion	Signaling pathway
Clark et al. (2006)	Sulforaphane (SFN)	Running mouse model	SFN reduces skeletal muscle injury and fatigue by inhibiting the PINK1/Parkin pathway	PINK1/Parkin
Botella et al. (2018)	Hongjingtian oral solution (RCOL)	Weight-bearing swimming mouse model	RCOL alleviates fatigue by inhibiting the PINK1/Parkin pathway	PINK1/Parkin
Wang et al. (2022)	Ginseng compound beverage	Weight-bearing swimming mouse model	The herbal drink alleviates fatigue by inhibiting the PINK1/Parkin pathway, but the dose effect needs to be further studied	PINK1/Parkin
Shang et al. (2018)	Astragalus seedling drink	Fatigue combined myocardial injury rat model	Astragalus membranaceus improved myocardial injury by inhibiting PINK1/Parkin pathway	PINK1/Parkin
Ma et al. (2023)	Grifolan	Chemotherapy-induced fatigue (CIF) mouse model	Arbutinan promotes PINK1/Parkin expression to restore mitochondrial autophagy homeostasis	PINK1/Parkin
Lei et al. (2019)	Yi Fei San Jie Wan	Swimming exhaustion cancer mouse model	Yi Fei San Jie Pills relieve excessive mitochondrial autophagy in skeletal muscle by inhibiting BNIP3/Nix pathway	BNIP3/Nix
Wu et al. (2023a)	Huangba extract	Exhausted exercise rat model	Huquba improves exercise performance by inhibiting FUNDC1/LC3B pathway	FUNDC1/LC3B
Toshiyuki et al. (2000)	Curcumin	T2DM rat model	Curcumin promotes mitochondrial autophagy by activating AMPK pathway	AMPK
Masayuki et al. (1997)	Herba Rhodiolae (RC)	Exhaustion motion model	By promoting the AMPK pathway, Red Jing Tian improves mitochondrial function and protects myocardial injury	AMPK
Dun et al. (2017)	Astragalan	Oxidative stress mouse model	Astragalus polysaccharide increased the activity of antioxidant enzymes and improved mitochondrial dysfunction	oxidative stress
Wang (2021)	Allyl tannin	Motion fatigue model	Aloe tannin improves fatigue by increasing mitochondrial autophagy and antioxidant capacity	oxidative stress

## 4 Conclusion and future perspectives

This review synthesizes evidence supporting the critical role of mitophagy’s in exercise-induced fatigue and discusses how natural compounds and pharmacological agents regulate this process. Mitophagy enhances antioxidant capacity while alleviating exercise fatigue through clearance of damaged mitochondria and oxidative stress reduction. Several interventions—including sulforaphane, Rhodiola-based formulations, and ginseng extracts—have demonstrated anti-fatigue effects through modulation of mitophagic pathways. These findings advance molecular understanding of exercise fatigue and establish a foundation for novel anti-fatigue therapeutics. Nevertheless, mechanistic aspects of mitophagy’s impact on exercise fatigue require further elucidation.

Animal models are fundamental for studying the mechanisms of exercise-induced fatigue and developing interventions. Rodents, such as SD/Wistar rats and ICR/BALB/c mice, are the most commonly used subjects. Classical approaches simulate physiological exhaustion through forced exercise, primarily using treadmill running or weight-loaded swimming protocols. However, these models have considerable limitations. Future directions include using gene-editing technologies to create models with specific genetic modifications and applying optogenetics or chemogenetics to precisely advance molecular-level insights.

Although existing research has addressed fatigue in specific diseases, such as mitochondrial dysfunction in Sjögren’s syndrome (Kurien et al., 2024), or focused on particular populations, such as those with chronic fatigue syndrome (Si et al., 2023), direct clinical studies involving healthy individuals or exercise-related fatigue remain scarce. Subsequent research should include targeted interventions, including examining how different exercise types or nutritional supplements affect autophagy and incorporate clinical trials to verify their effectiveness.

Future studies should prioritize multidisciplinary approaches that integrate cellular, molecular, and systemic perspectives to clarify context-specific mitophagy mechanisms. Well-controlled investigations are needed to determine how different exercise modalities and natural compounds precisely influence mitophagic activity, and to identify their direct molecular targets. Such efforts will help translate these findings into targeted anti-fatigue interventions.
